# Reevaluating Routine Post-Biopsy Chest X-Rays After CT-Guided Lung Biopsy: Incidence of Pneumothorax and Value of Symptom-Based Monitoring

**DOI:** 10.3390/jcm14144867

**Published:** 2025-07-09

**Authors:** Rosa Alba Pugliesi, Ina Schade, Amina Benchekroun, Roua BenAyed, Andreas Mahnken, Nour Maalouf, Jonas Apitzsch

**Affiliations:** 1 Section of Radiology, Department of Biomedicine, Neuroscience and Advanced Diagnostics (BiND), University of Palermo, Via del Vespro 129, 90127 Palermo, Italy; 2 Department of Thoracic Surgery, Helios Hospital Pforzheim, 75175 Pforzheim, Germany; ina.schade@helios-gesundheit.de; 3 Department of Radiology and Nuclear Medicine, Helios Hospital Pforzheim, 75175 Pforzheim, Germany; amina.benchekroun@hotmail.com (A.B.); roua.benayed@helios-gesundheit.de (R.B.); jonas.apitzsch@helios-gesundheit.de (J.A.); 4 Department of Diagnostic and Interventional Radiology, University Hospital of Marburg, 35043 Marburg, Germany; andreas.mahnken@staff.uni-marburg.de; 5 Department of Radiology and Nuclear Medicine, Tübingen University Hospital, 72076 Tübingen, Germany; nourmaalouff@gmail.com

**Keywords:** pneumothorax (PTX), CT-guided lung biopsy, chest drainage, post-procedural complication, follow-up imaging

## Abstract

**Background/Objectives**: The aims of this study were to re-evaluate the necessity routine post-biopsy chest X-rays by evaluating the incidence, timing, and clinical relevance of pneumothorax (PTX) following CT-guided lung biopsies, and to determine whether symptom-based monitoring can safely replace routine imaging. **Methods**: This retrospective, single-center study included 112 patients (mean age: 69.3 years; 55% male) who underwent CT-guided lung biopsy between 9 January 2020 and 16 April 2025. PTX occurrence was assessed both intraprocedurally and during follow-up within 7 days. The primary outcome was the development of PTX; secondary outcomes were chest drainage need and delayed PTX identification. Logistic regression analysis and descriptive statistics were used to identify predictors and associations. **Results**: Intra-procedural PTX occurred in 43.8%, of whom 10.7% required immediate drainage. Clinically silent PTX that did not require intervention occurred in 25.9%. Routine chest X-rays were obtained 4 h post-biopsy for all patients. Importantly, no asymptomatic patient required any intervention. These results suggest that routine 4 h imaging may be unnecessary in the absence of symptoms. **Conclusions**: PTX is frequent after CT-guided lung biopsy but is often asymptomatic and self-limiting. The absence of clinically significant findings among asymptomatic patients supports a shift toward symptom-based monitoring. Implementing this strategy may help reduce unnecessary post-biopsy imaging and optimize healthcare resource utilization without compromising patient safety.

## 1. Introduction

CT-guided lung biopsy is an essential diagnostic tool for evaluating pulmonary lesions, but it carries a well-known risk of complications, most notably pneumothorax (PTX) [1,2]. Whereas some presentations of PTX require urgent intervention, such as chest drainage, others are clinically silent and resolve spontaneously without intervention [3,4]. To ensure the early diagnosis of asymptomatic PTX, it has been routine to screen patients after the biopsy procedure with a chest X-ray, although there has been debate about whether this is necessary in all patients [5,6].

With the goal of eliminating unwarranted imaging and optimizing the use of healthcare resources, this study aims to reassess the need for routine post-procedure chest X-rays after CT-guided lung biopsy [5,7]. Specifically, it examines the incidence of PTX, its association with demographic and procedural factors, the need for chest drainage, and the potential value of symptom-based monitoring in guiding post-biopsy care.

## 2. Materials and Methods

**Figure 1:**
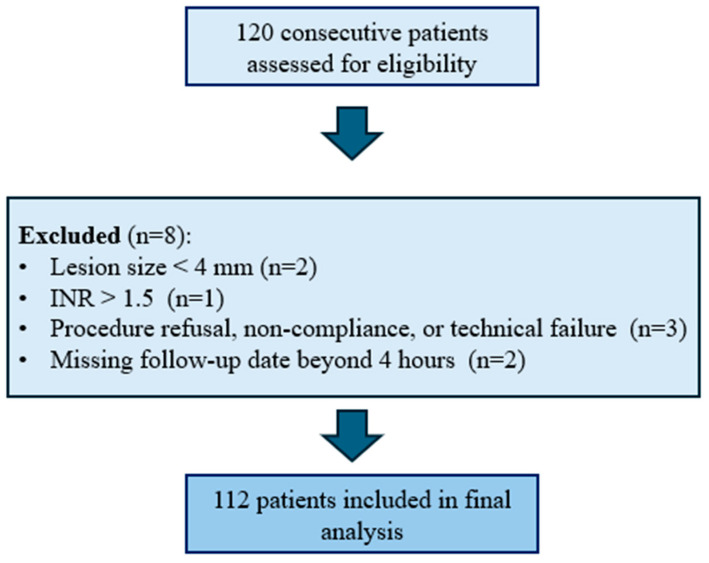
Flowchart of patient inclusion and exclusion.

This retrospective, single-center study initially enrolled 120 consecutive patients (mean age: 69.28 years, range: 49–90 years; 55.0% male) scheduled for CT-guided lung biopsy between 9 January 2020 and 16 April 2025. Of these, 8 patients were excluded based on predefined exclusion criteria (Figure 1), resulting in a final cohort of 112 patients for analysis. All patients provided written informed consent at least 24 h before the procedure. Ethical approval for this study was provided by the local ethics committee (F-2021-038).

All patients were discussed in a multidisciplinary tumor board involving oncology, pathology, radiology, pulmonology, and thoracic surgery.

Biopsies were performed for diagnostic purposes in patients presenting with indeterminate etiology lung lesions, including suspected lung primary tumors, metastases, or atypical infections.

Only intrapulmonary parenchymal lesions were eligible; no mediastinal masses or lymph node biopsies were included. All lesions were unsuitable for bronchoscopy owing to peripheral location, very small size, or lack of bronchial access.

Patients with pre-existing lung conditions predisposing to pneumothorax (e.g., emphysema or cystic lung disease) were retained in the study and are captured under the COPD (Chronic Obstructive Pulmonary Disease) variable.

CT-guided lung biopsies were performed using an 18G semi-automatic Tru-Cut needle and a 17G trocar (Möller Medical GmbH, Fulda, Germany), with the patients in a prone or supine position, depending on the location of the lesion. During the procedure, the angle of the needle relative to the pleural surface on the left/right plane was measured and later correlated with the incidence of PTX as either an intra-procedural or early post-procedural complication.

The primary outcome was the occurrence of post-procedure PTX as revealed by imaging (chest X-ray) 4 h after CT-guided biopsy. The secondary outcomes included the need for chest drainage at the time of procedure and the detection of PTX on follow-up imaging in <7 days from the procedure.

A standardized symptom-monitoring protocol was applied to identify early and delayed complications. Monitored symptoms included chest pain, dyspnea, new onset of tachycardia, new onset of cough, and reduced or absent breath sounds on auscultation. Pneumothorax (PTX) was the primary complication being monitored.

The monitoring was performed by radiologists, attending physicians, and nurses. The patients were observed by radiology for 10–20 min post-intervention. A written report was prepared, and an oral handover was made immediately to the attending physician.

Monitoring intervals included directly after the procedure, before transfer to the ward, at 1 h, at 4 h post-biopsy, during evening ward rounds, and again during morning rounds. These clarifications have been incorporated to enhance the reproducibility and clinical relevance of our findings.

Among the 20 patients with delayed pneumothorax, 8 were symptomatic. Six of these required drainage, while none required hospital readmission.

### 2.1. Inclusion and Exclusion Criteria

This observational study included patients who underwent CT-guided lung biopsies for the histopathological evaluation of indeterminate lung lesions unsuitable for bronchoscopic biopsy. Exclusion criteria included lesions with a diameter < 4 mm, International Normalized Ratio (INR) of >1.5, an inability to follow instructions or a refusal to undergo the procedure, missing follow-up data beyond 4 h, prior recent pneumothorax or existing pleural drainage, and aborted or technically failed procedures. A flowchart of included and excluded patients is shown in Figure 1.

### 2.2. Biopsy Needle

The 18G biopsy needle is a semi-automated Tru-Cut design with a central sharp stylet surrounded by a hollow cylindrical sheath. Tru-Cut design needles are widely used in lung diagnostic biopsies due to their ease of use and procedural efficiency [8].

### 2.3. Trocar

The 17G trocar (Möller Medical GmbH, Fulda, Germany) facilitates multiple biopsies of the same intrapulmonary lesion with only a single pleural passage, as it acts as a guide for the 18G needle.

### 2.4. Biopsy Protocol

Selective CT scanning of the region of interest (as determined from preintervention scans) was obtained at the end-expiration breath-hold. All patients were scanned in prone or supine position in a 64-slice spiral CT Scanner (Siemens Somatom Edge, Siemens, Forchheim, Germany) with no i.v. contrast enhancement. Native spiral CT of the thorax was obtained with adaptive tube modulation in expiration. A reconstruction in 2 mm slice thickness with 1 mm increment was performed in both lung window and soft tissue window techniques for intervention planning. During intervention, sequential CT was used in 2.4 mm slice thickness without increment at 20 mAs, applying both lung window and soft tissue window techniques. This protocol was used for both the guidance phase and the post-procedural control scans. The control imaging was performed using a low-dose, non-contrast chest scan in end-expiration to assess for post-biopsy complications. We ensured preparation for intervention, prepared and cleaned the puncture site, used local anesthetic (1% mepivacaine), and created a minute incision into the skin. We inserted the coaxial needle within the lesion margin and passed the 18G needle through the trocar to collect a sample for biopsy. We fixed the obtained samples using formaldehyde and subjected them to histopathological analysis.

**Figure 2:**
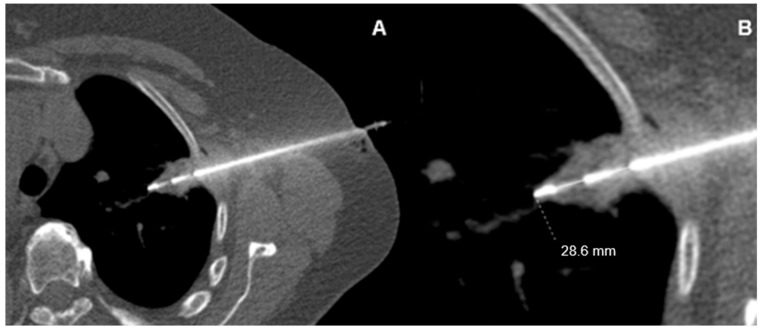
CT-guided lung biopsy in a pleura-adherent lesion. (**A**) Axial CT image showing a pleura-adherent lesion during biopsy. (**B**) Although the lesion contacts the pleura (0 mm distance), the needle penetrates 28.6 mm into the lung. This deep positioning ensures the biopsy cylinder is fully intrapulmonary, avoiding a punching defect in the pleura and minimizing pneumothorax risk.

After removing the biopsy needle and closure of the trocar puncture with a sterile patch, a low-dose non-contrast control chest scan in end-expiration was performed to assess for potential complications of intervention. This imaging protocol enhances transparency and reproducibility and may be used as a technical reference in similar clinical settings (Figure 2). No blood patches or multiple pleural passes were employed. There was never more than a single pleural passage associated with the trocar needle.

### 2.5. Statistical Analysis

Statistical analyses were conducted to evaluate five primary outcomes: (1) the association between patient position during the CT-guided lung biopsy (prone vs. supine) and PTX occurrence, (2) the influence of sex on the risk of PTX, (3) the correlation between age and PTX risk, (4) the association between follow-up imaging and the detection of delayed PTX, and (5) the relationship between positioning and the need for CT-guided drainage. Patients were categorized based on the development of PTX, as assessed on follow-up chest X-ray 4 h after biopsy. All patients followed a standardized imaging protocol: A routine chest X-ray was systematically performed approximately 4 h after CT-guided lung biopsy to identify immediate or early PTX. Subsequent follow-up imaging, either chest X-ray or CT scan, was performed selectively based on clinical symptoms or, in some cases, as routine surveillance within 7 days. Therefore, while 4 h imaging was applied consistently across the entire cohort, the follow-up imaging protocol beyond 4 h exhibited some variability depending on clinical judgment and symptom presentation.

Categorical variables, including sex, positioning, and follow-up imaging status, were analyzed using Fisher’s exact test. Continuous variables, such as age, were assessed using Pearson’s correlation coefficient. Variations in the frequency with which PTX occurred over the study period of 5 years and 3 months were established using the chi-square test for trends. Kaplan–Meier survival plots were constructed to display cumulative PTX-free rates.

All statistical tests were two-tailed with a significance level of *p* < 0.05. Statistical analysis was performed using R software (version 4.4.2).

## 3. Results

The mean age of the final cohort of 112 patients was 69.3 ± 9.2 years (range 42–89 years). There were 62 males and 50 females, and no statistically significant difference in age was found between those who developed pneumothorax (PTX) and those who did not (*p* = 0.715).

Lung lesions were in all five lobes: 28 in the right upper lobe, 10 in the right middle lobe, and 29 in the right lower lobe; 29 in the left upper lobe and 24 in the left lower lobe. The distribution indicates a fairly even involvement of both lungs with very slightly increased frequency in the upper lobes. Lesions affected central and peripheral regions but were predominantly peripherally based on CT-guidance planning and trajectory. No mediastinal lesions were included.

Table 1 summarizes these demographics together with COPD prevalence, biopsy position, pneumothorax rates, drainage requirements, and follow-up imaging data, allowing the reader to appreciate the key findings at a glance.

### 3.1. Influence of Patient Position (Prone vs. Supine) on Pneumothorax

To assess whether patient position during the CT-guided lung biopsy affected the likelihood of PTX, we performed Fisher’s exact test based on a contingency table (Table 2).

The analysis yielded *p* = 0.852, with an estimated odds ratio of 0.91 (95% CI: 0.41–2.05). This suggests no statistically significant association between patient position and the development of PTX (the shaded areas in the figure represent 95% confidence intervals). Therefore, we concluded that placing the patient in a prone or supine position did not influence the incidence of PTX in our patient cohort.

**Figure 3:**
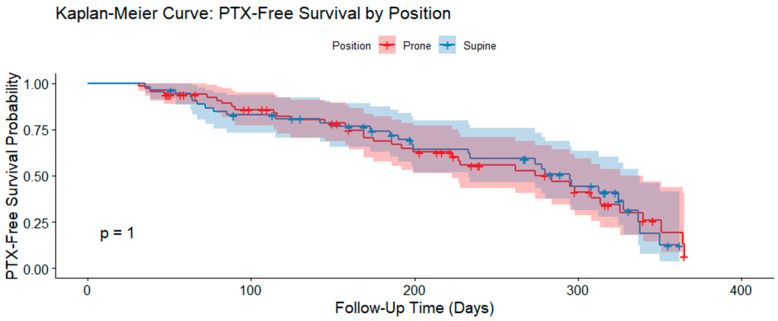
Kaplan–Meier curves illustrating pneumothorax (PTX)-free survival probability by patient position (prone vs. supine) over time. Shaded areas represent 95% confidence intervals. No statistically significant difference was observed between the groups (*p* = 1).

Figure 3 presents Kaplan–Meier curves of PTX-free survival for the prone and supine groups. These are the curves representing the probability of being free of PTX at any point in time for each group. The analysis revealed no statistically significant difference in PTX-free survival between the two groups (*p* = 1), confirming that patient position had no effect on the timing or risk of PTX development during the follow-up period. These findings demonstrate that patient position and follow-up imaging do not significantly impact the incidence and timing of PTX after CT-guided lung biopsy. This, in turn, suggests the value of post-procedural symptom-based monitoring of the need for PTX drainage for optimizing resource utilization without compromising patient outcome.

### 3.2. Influence of Sex on Pneumothorax Occurrence

We explored whether sex was related to PTX occurrence using Fisher’s exact test based on a contingency table (Table 3).

Fisher’s exact test resulted in *p* = 1.000, indicating no significant association between sex and PTX. The odds ratio of 1.03 (95% CI: 0.44–2.37) suggests no sex-related difference in the risk of PTX. These results demonstrate that sex does not represent a predictive factor for PTX following CT-guided lung biopsy. Therefore, no risk stratification or procedural adjustment is warranted based on patient sex.

### 3.3. Correlation Between Age and Pneumothorax Risk

The correlation between patient age and risk of PTX after CT-guided lung biopsy was evaluated through Pearson’s correlation analysis. As aligned with the research aim to establish demographic predictors of PTX following this procedure, the analysis gave a correlation coefficient of −0.038 and a related *p*-value of 0.715. The findings indicate an extremely weak, non-significant negative correlation between age and PTX occurrence. In this population, whose mean age was 69.3 years, age did not prove to be an important risk factor for the development of either immediate or delayed PTX.

This suggests that enhanced post-procedure surveillance or routine follow-up imaging should not be selected simply because of the advanced age of the patient. The lack of a strong association between age and PTX reinforces that a symptom-based monitoring protocol may be preferable over age-driven follow-up strategies.

### 3.4. Association Between Follow-Up Imaging and Detection of Delayed Pneumothorax

We examined whether patients who underwent follow-up imaging within 7 days were more likely to present delayed PTX, as shown in Table 4.

**Figure 4:**
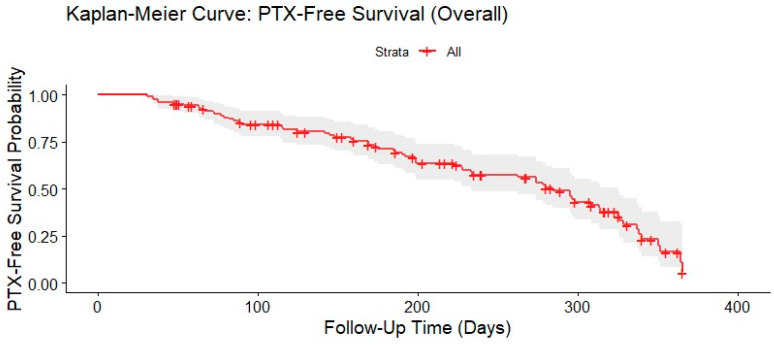
Kaplan–Meier curve illustrating pneumothorax (PTX)-free survival probability for the whole cohort over time. Shaded areas represent 95% confidence intervals.

A Kaplan–Meier curve of the whole cohort’s PTX-free survival (Figure 4) was prepared to illustrate the cumulative probability of freedom from PTX during the follow-up period after CT-guided lung biopsy. This analysis served the study’s primary purpose by demonstrating the timing and occurrence of both early and late PTX events, particularly in determining whether routine post-procedure chest X-rays are needed. The survival curve showed a gradual decline in the probability of remaining free from PTX over time, reflecting the occurrence of clinically inapparent and delayed PTX identified during follow-up imaging. After excluding cases with unknown PTX status, Fisher’s exact test yielded *p* = 0.2969, with an estimated odds ratio of 2.09 (95% CI: 0.61–9.26). Although patients who underwent follow-up imaging during the 7 days appeared more likely to develop delayed PTX, the association was not statistically significant. The wide confidence interval indicated substantial uncertainty, which could potentially be explained by the small number of observed cases of delayed PTX. Overall, the Kaplan–Meier curve provided additional evidence to support the application of a symptom-based monitoring approach to reduce unnecessary imaging without compromising patient safety.

### 3.5. Association Between Patient Position During Biopsy Procedure and CT-Guided Drainage Placement

Finally, we investigated the association between patient position and the need for CT-guided drainage, using a contingency table (Table 5).

Both Fisher’s exact test (*p* = 0.3743) and the chi-square test with Yates’ correction (*p* = 0.4475) confirmed that there was no statistically significant association. These findings support the conclusion that patient positioning during the biopsy does not significantly influence the need for CT-guided drainage, and therefore this parameter should not alter procedural planning.

### 3.6. Histological Outcomes

Histopathological evaluation was successfully completed in 109 out of 112 patients (97.3%), while 3 cases (2.7%) were non-diagnostic due to either insufficient material or the absence of tumor cells. Among diagnostic cases, malignancies accounted for 84.8% (95/112). The most frequent subtype was adenocarcinoma, including lepidic, poorly differentiated, and pulmonary variants, being the most frequent subtype, identified in 44 patients (39.3%). Non-small-cell lung cancer not otherwise specified (NSCLC NOS) was diagnosed in 15 patients (13.4%), followed by squamous-cell carcinoma in 12 patients (10.7%) and small-cell lung cancer (SCLC) in 6 patients (5.4%).

Neuroendocrine tumors, including carcinoids and atypical variants, were found in four patients (3.6%), and other primary pulmonary malignancies (e.g., sarcoma, carcinosarcoma, fibrous tumor, and lymphoma) were diagnosed in four patients (3.6%). Pulmonary metastases from extrapulmonary primaries—mainly originating from renal cell carcinoma (two cases), breast carcinoma (two), endometrial carcinoma (one), malignant melanoma (one), and mammary carcinoma (one)—were observed in seven patients (6.3%). Additionally, three cases (2.7%) were classified as other or unspecified carcinomas, including ambiguous diagnoses labeled as “carcinoma” or “lung cancer” without histologic subtyping.

Benign or inflammatory lesions were reported in 16 patients (14.3%), including organized pneumonia (3 cases; 2.7%), pneumonia (3; 2.7%), fibrosis (3; 2.7%), granulomatous disease such as sarcoidosis or rheumatoid arthritis-related granulomas (2; 1.8%), abscesses (2; 1.8%), chronic inflammation or heart failure-associated changes (2; 1.8%), and chondrohamartoma (1; 0.9%).

**Figure 5:**
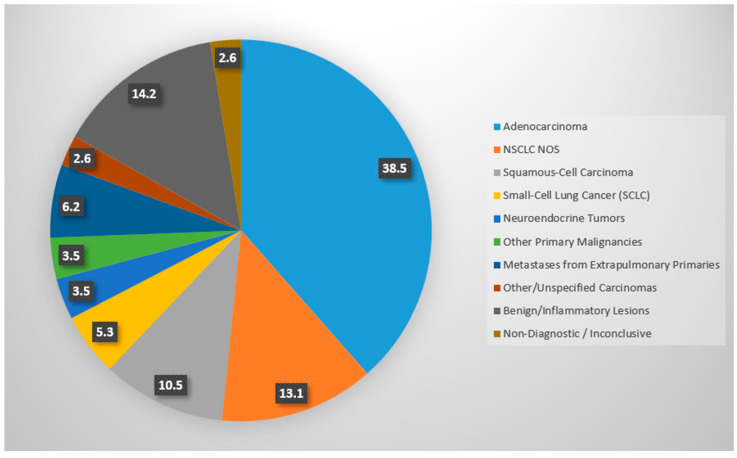
Pie chart illustrating the distribution of histological diagnoses among the study cohort. NSCLC NOS indicates non-small-cell lung carcinoma not otherwise specified. The Benign/Inflammatory group comprises non-neoplastic findings, including organized pneumonia, chronic inflammatory changes, and pulmonary fibrosis.

The distribution of histological findings is depicted in Figure 5.

## 4. Discussion

This study provides a clinically relevant and practical assessment of risk factors for PTX in patients undergoing CT-guided lung biopsies, offering new insights that can inform procedural planning and post-biopsy surveillance protocols. Of particular interest, our findings contribute to the growing debate over the use of post-procedural imaging, supporting a selective, symptom-based approach.

Our study assessed both patient-related and procedure-related factors in relation to the incidence of PTX. Specifically, we systematically examined the roles of patient positioning, sex, age, and follow-up imaging protocols in the development of PTX and the requirement for drainage.

Contrary to prior expectations, patient position (prone vs. supine) did not show a statistically significant relationship with PTX occurrence. Although some earlier studies suggested that prone positioning may limit lung movement and reduce lesion depth, potentially lowering PTX risk [9,10,11], our data did not confirm this. The odds ratio (0.91) and Kaplan–Meier analysis also indicated no meaningful correlation between patient position and PTX development or timing. Given the high *p*-values in our correlational analysis, we assert that there is no association in our dataset. This contrasts with a large meta-analysis of over 23,000 patients, which reported that lateral decubitus positioning (biopsied lung down) significantly reduced PTX risk [12,13]. We could not replicate this finding due to the absence of lateral decubitus cases in our sample, highlighting how procedural variability and study design influence outcomes.

Sex was similarly unassociated with PTX development, with comparable rates among males and females. This aligns with previous research reporting no sex-related differences in PTX risk after lung biopsy [14].

Age, another potential predictor of post-biopsy complications, also showed no significant association with PTX risk in our cohort. This is consistent with prior studies [15,16,17] and suggests that older age alone may not necessitate intensified post-procedural monitoring, further supporting the shift toward symptom-based rather than demographic-based follow-up protocols.

While a higher number of delayed PTX cases were observed in patients who received follow-up imaging within 7 days, the association was not statistically significant. The broad confidence interval reflects limited statistical power due to the small number of delayed PTX cases. Importantly, all patients underwent a standardized 4 h post-procedure chest X-ray, whereas subsequent imaging was selectively guided by clinical need or institutional routine, introducing follow-up variability beyond this initial timeframe.

Our findings echo those of prior large-cohort and systematic reviews that concluded that most delayed PTX cases are either clinically insignificant or symptomatic [18,19,20]. This supports a shift toward individualized, symptom-guided imaging in asymptomatic patients.

In terms of real-life clinical impact, our findings indicate that there ought to be more individualized, cost-effective post-biopsy management. Specifically, asymptomatic patients following a benign 4 h X-ray might safely be released with no further routine imaging, which may reduce unnecessary radiation exposure, workload burden, and healthcare costs. This is one of the few studies to assess both PTX-free survival and chest tube insertion in conjunction with clinical symptom evaluation. Notably, none of the asymptomatic patients in our cohort required drainage, corroborating earlier reports that clinical observation in this subgroup is safe [21,22].

Recent meta-analyses have emphasized strategies to minimize PTX risk. A pooled analysis of 4116 patients demonstrated that autologous blood patching during needle withdrawal significantly reduced both PTX rates (RR = 0.65) and chest tube insertion (RR = 0.45) [23]. Similarly, the PEARL protocol, which includes procedural positioning, track sealing, and symptom-based imaging, reduced PTX from 37% to 16% and chest tube placement from 13% to 1%, even among high-risk emphysema patients [24].

Taken together, our results and the recent literature reinforce the principle that routine imaging in asymptomatic patients offers limited additional clinical benefit. A structured symptom-guided monitoring approach, in combination with procedural refinements, is both safe and resource-conscious [13,23,24].

Finally, patient positioning during biopsy did not influence the need for CT-guided drainage. This suggests that drainage requirements are driven by other procedural or anatomical factors [25].

Limitations of our study include its retrospective, single-center design and the possibility of underestimating complication rates due to the involvement of highly experienced operators. Furthermore, subgroup analyses may have been underpowered, and variability in follow-up imaging could have introduced detection bias for delayed PTX. Additionally, while major symptoms such as dyspnea and chest pain were routinely assessed, the formal documentation of parameters such as respiratory rate, oxygen saturation, or standardized symptom scores was inconsistent. This limits generalizability and emphasizes the need for prospective studies using structured clinical monitoring.

Future research should aim to validate our findings in multicenter prospective cohorts with standardized symptom-based follow-up protocols and formalized risk assessment tools. Furthermore, the exploration of adjunctive techniques such as track sealing, patient-specific positioning strategies, or automated symptom monitoring could help refine best practices for minimizing PTX risk.

Our results contribute meaningfully to the ongoing effort to balance patient safety with responsible healthcare resource utilization [26]. By demonstrating that patient age, sex, and position do not independently predict PTX, and that asymptomatic patients rarely require intervention, we support the development of personalized, symptom-driven monitoring pathways that reflect the evolving priorities of value-based clinical care.

Despite these limitations, the consistency of our findings with high-quality cohort and meta-analytic studies strengthens their external validity [27,28,29].

## 5. Conclusions

This study provides helpful evidence to support a more efficient and patient-focused post-biopsy practice. Through the systematic evaluation of pneumothorax outcomes after CT-guided lung biopsy in a well-defined patient cohort, we demonstrated that routine post-procedural imaging, particularly in asymptomatic patients, may have limited clinical utility. The findings of our research emphasize the feasibility and safety of a symptom-based follow-up strategy, with potential workflow reduction, radiation sparing, and healthcare savings without a negative impact on patient outcomes. Importantly, this research contributes to the evolving paradigm in interventional radiology, in alignment with international efforts towards the creation of evidence-based, risk-stratified follow-up guidelines. While additional prospective studies are needed to confirm these findings across diverse clinical settings, our results offer a compelling argument for reevaluating current imaging routines and adopting more personalized care pathways in the post-biopsy setting.

## Figures and Tables

**Table 1:** Summary of patient characteristics and outcomes. Overview of key clinical and procedural variables in 112 patients undergoing CT-guided lung biopsy, including age, sex, lesion location by lobe, COPD status (including emphysema and cystic changes), biopsy position, pneumothorax occurrence, follow-up imaging, and drainage requirements.

Variable	Value
Total number of patients analyzed	112
Mean age (years)	69.3
Sex	Male: 62 (55.4%) Female: 50 (44.6%)
Location (Lobe)	
Right upper lobe	28 (25.0%)
Right middle lobe	10 (8.9%)
Right lower lobe	29 (25.9%)
Left upper lobe	29 (25.9%)
Left lower lobe	24 (21.4%)
COPD	34 (30.4%)
Patient position during biopsy	Prone: 52 (46.4%) Supine: 60 (53.6%)
Pneumothorax detected (any)	49 (43.8%)
Clinically inapparent pneumothorax	29 (25.9%)
Pneumothorax requiring drainage	12 (10.7%)
Follow-up imaging within 7 days	75 (67.0%)
Pneumothorax on follow-up imaging (delayed PTX)	20 (17.9%)
Drainage placement during CT intervention	10 (8.9%)
Drainage placement during follow-up	8 (7.1%)

**Table 2:** Distribution of pneumothorax (PTX) occurrence in patients placed in a prone or supine position.

Position	No PTX	PTX
Prone	31	21
Supine	40	20

**Table 3:** Distribution of pneumothorax (PTX) occurrence by sex.

Sex	PTX = 0	PTX = 1
Male	39	23
Female	30	10

**Table 4:** Distribution of patients based on follow-up imaging within 7 days and identification of pneumothorax (PTX) in follow-up imaging.

Follow-Up Imaging Within 7 Days	PTX on Follow-Up (Yes)	PTX on Follow-Up (No)
Yes (n = 75)	18	57
No (n = 37)	2	35

**Table 5:** Distribution of need or no need for drainage placement by patient position (prone vs. supine) during the procedure.

Position	Drainage Placement (No)	Drainage Placement (Yes)
Prone	38	14
Supine	46	14

## Data Availability

The data presented in this study are openly available in FigShare (https://doi.org/10.6084/m9.figshare.29094362).
